# Historical Environment Is Reflected in Modern Population Genetics and Biogeography of an Island Endemic Lizard (*Xantusia riversiana reticulata*)

**DOI:** 10.1371/journal.pone.0163738

**Published:** 2016-11-09

**Authors:** Iris A. Holmes, William J. Mautz, Alison R. Davis Rabosky

**Affiliations:** 1 Department of Ecology and Evolutionary Biology, University of Michigan, 1109 Geddes Ave, Ann Arbor, MI, 48103, United States of America; 2 Department of Biology, University of Hawaii at Hilo, Hilo, HI, 96720, United States of America; 3 Department of Ecology and Evolutionary Biology, University of California Santa Cruz, 1156 High St., Santa Cruz, CA, 95064, United States of America; University of Regina, CANADA

## Abstract

The restricted distribution and isolation of island endemics often produces unique genetic and phenotypic diversity of conservation interest to management agencies. However, these isolated species, especially those with sensitive life history traits, are at high risk for the adverse effects of genetic drift and habitat degradation by non-native wildlife. Here, we study the population genetic diversity, structure, and stability of a classic “island giant” (*Xantusia riversiana*, the Island Night Lizard) on San Clemente Island, California following the removal of feral goats. Using DNA microsatellites, we found that this population is reasonably genetically robust despite historical grazing, with similar effective population sizes and genetic diversity metrics across all sampling locations irrespective of habitat type and degree of degradation. However, we also found strong site-specific patterns of genetic variation and low genetic diversity compared to mainland congeners, warranting continued special management as an island endemic. We identify both high and low elevation areas that remain valuable repositories of genetic diversity and provide a case study for other low-dispersal coastal organisms in the face of future climate change.

## Introduction

Islands, due to their isolation, often support suites of highly endemic species and contain some of the most threatened habitats in the world [1, 2, 3]. Islands face similar threats to mainland habitats, such as habitat degradation and global climate change [4, 5]. However, island endemics are also sensitive to loss of genetic diversity and stochastic population fluctuations caused by the small size and isolation of their habitat [6, 7]. Insular species can be particularly vulnerable to invasive species, through predation, competition, or habitat destruction [8, 9]. Despite these challenges, islands also offer the opportunity to completely eradicate invasive species, which is not often feasible on continental scales [10, 11, 12]. Improving conservation techniques for island endemics is a goal of global importance, as island species often represent unique phenotypic or genetic diversity that is of high priority to wildlife managers.

Maintaining genetic connectivity and genetic diversity are central concerns in conservation genetics [13, 14, 15]. Species with relatively low vagility present a particular challenge because genes may not move freely despite having no discernible barriers to gene flow [16, 17]. In such species, loss of a small area of habitat can represent a substantial reduction in the overall genetic diversity and evolutionary potential of the population, if that habitat hosts a genotype that has not diffused through the population [18]. As such, thorough survey of the available genetic diversity is a critical step in conservation planning for the maintenance of that diversity. Moreover, concentrations of genetic diversity can form in sections of habitat that were formerly connected to large populations that have recently contracted, as simulation studies indicate that many generations must pass at low population size before genetic diversity is lost [19]. Identifying these hotspots may be facilitated by both genetic approaches and knowledge of the historical extent of the species.

### San Clemente Island biogeography

San Clemente Island, like the other seven California Channel Islands, formed as the Farallon plate subducted under the North American plate during the Miocene [20, 21, 22]. At that time, San Clemente Island may have been connected to Baja California, far to the south of its current position [23, 24]. After the Miocene, the subduction zone moved away from southern California, and the counterclockwise rotation of the Pacific Plate led to the formation of a field of roughly parallel faults with northwestward movement, one of which continues to push San Clemente island north, west, and upward [21, 25]. The tectonic uplift resulted in the carving of twenty sea terraces currently visible on the west side of the island [26]. The oldest terrace, currently 300 meters above sea level, was carved 1.25 mya, while the youngest, five meters above sea level, was carved 90,000 years ago [26].

At the time of the last glacial maximum (LGM), global sea levels were 120 meters lower than current levels [27]. The land exposed by lowered sea levels increased the area of San Clemente Island to 1.7 times its current size, expanding the total land area available to the island organisms. Most of the exposed land was on the western side of the island. By 2000 YA, the global sea level had stabilized close to its current position [27]. This large, geologically recent habitat reduction may have affected the current population genetic patterns of terrestrial species on the island.

### *Xantusia riversiana* and its phylogenetic relationships

**Fig 1:**
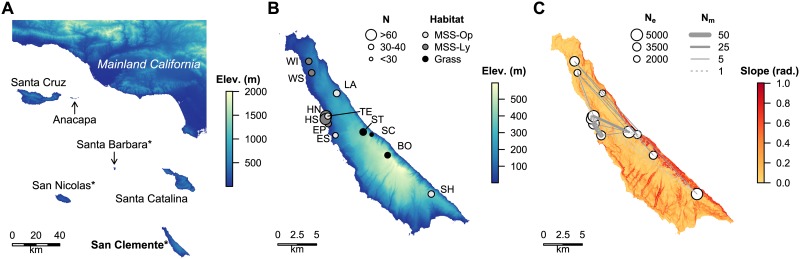
Sampling locations and characteristics. a Relief map of the Channel Islands and mainland California. Island names with asterisks are inhabited by *Xantusia riversiana*, with our study island in bold. b Relief map of San Clemente Island showing collection locations colored by habitat type and scaled by sample size (N). c Map of San Clemente Island colored by slope steepness and showing dispersal rates among collection locations. Points are scaled by effective population size (N_e_) and lines by number of migrants per generation (N_m_).

*Xantusia* is a genus of secretive lizards that occurs in the western US and Mexico [23]. *Xantusia riversiana* is the largest member of the genus at 80–110 mm snout-vent length and is a classic example of island gigantism [28]. A true island endemic, it only occurs on three of the California Channel Islands (asterisks in Fig 1a): San Clemente Island, Santa Barbara Island, and San Nicolas Island (where it is considered a separate subspecies, *Xantusia riversiana riversiana*). Night lizards tend to exhibit high site fidelity, with adults spending their 20–30 year life spans within small territories. Over six months, 45% of marked *X*. *riversiana* on San Clemente Island were found at their original capture locations, while the others dispersed an average of 3 m [28]. As young *Xantusia* rarely disperse far from their natal site (an average of 4.2 meters in *Xantusia vigili*s; [29]), their population genetic distribution reflects demographic events both ancient and modern [23].

*Xantusia riversiana* may be the sister species to all other members of the genus, from which it diverged approximately 14 to 16 MYA ([23]; but see [30] and [31] for alternative tree topologies and divergence time estimates). At that time, the flora of Baja California was primarily tropical deciduous forest, with evergreen broadleaf forest present in riparian areas [32]. Fossil tree species from areas in Baja California near the putative attachment point of Miocene San Clemente Island most closely resemble extant tropical dry forests on the west coast of Mexico, which is within the current range of *Lepidophyma*, the sister genus to *Xantusia* [23]. If this phylogenetic hypothesis is correct, the ancestral *Xantusia* had split from *Lepidophyma* and occurred in Baja California 20 million years ago. San Clemente Island’s separation from the mainland may have divided the ancestral *X*. *riversiana* from the rest of the *Xantusia* lineage, which subsequently speciated on the mainland. Alternatively, the islands could have been colonized by dispersal over water after they separated from the mainland. Both San Nicolas and Santa Barbara islands were completely submerged at times during the Miocene, while San Clemente Island remained above water, making it likely that the San Clemente population is ancestral to the populations on other islands [22, 25].

### Human impacts on San Clemente Island

San Clemente Island’s vegetation was severely damaged by invasive goats between their introduction in 1875 [33] and removal in 1991 [34]. In some areas, goat herbivory catalyzed a change from the endemic marine sage scrub to grassland that is largely composed of invasive Mediterranean grasses. These disturbances are expected to have had negative conservation consequences for *X*. *riversiana* by removing prime habitat that supports the highest density of lizards [35], but to date there has been no investigation of genetic population structure across habitat types to help inform management decisions. The native flora has been rebounding in the absence of the goats [33].

In this paper, we assess the population genetics and historical biogeography of the San Clemente Island Night Lizard, *Xantusia riversiana reticulata*, a species endemic to the Channel Islands of California, USA (S1 Fig and Fig 1a). First, we use DNA microsatellite markers augmented by genome-scale SNP data to estimate population genetic diversity, structure, connectivity, and stability in order to identify populations of particular conservation genetic importance. Second, we use the island’s history to examine the relative genetic impacts of two habitat disruptions: 1) the drastic reduction in size of the total island due to post-Pleistocene sea level rise, which occurred around 15,000 years ago, and 2) habitat degradation due to introduced goat herbivory, which began 150 years ago. Together, these analyses provide important insight into past demographic history, contextualize future management priorities and strategies, and offer a case study for comparison to other low-dispersal island organisms that have had significant habitat disturbance due to invasive species.

## Methods

### Ethics Statement

The U.S. Department of the Nave supported field collection and granted access to San Clemente Island, and the U.S. Fish and Wildlife Service issued scientific collecting permits to WJM. All methods were approved by the Chancellor’s Animal Research Committee (protocol #Sine0002-1) at the University of California, Santa Cruz.

### Tissue collection and site descriptions

We collected 530 tissue samples from 12 sites across San Clemente Island between 2005–2007 (Fig 1b; see Table 1 for site-specific sample sizes). We captured lizards through a combination of rock turn surveys and a previously established grid of pitfall traps [35]. At each capture, we measured the mass, snout-vent length (SVL), tail condition (broken, regenerated, intact), and determined the lizard’s sex by shining a light through the lizard’s tail base to visualize hemipenes in juvenile males [36] and by assessing hemipenal bulging to identify adult males. Because population monitoring was ongoing, some captured individuals had already been toe-clipped during previous population sampling, in which case the lizard’s clip combination and status as a recapture were noted. We toe-clipped each newly captured individual with a unique combination for future identification. We took a 0.5–1 cm piece of tail tissue (stored in 95% ethanol) from every lizard for genetic analyses. We did not use analgesia, as toe-clipping with sharp scissors does significantly not increase lizard stress-hormone levels and the increased manipulation necessary to use analgesia could potentially further stress the lizards [37]. We immediately released all lizards at their exact location of capture and recorded the latitude and longitude coordinates of each location using Magellan^®^ eXplorist^®^ 300 GPS units.

**Table 1:** Number of genotyped individuals (N), theta values, estimated effective population sizes (N_e_), and demographic trap capture rates for 12 sampling locations on San Clemente Island.

Site	N	θ ^1^	θ ^2^	mean θ	N_e_	Lizards/trap/ day^2^
BO	32	NA	12.60	12.60	3150.0	0.538*
EP	37	24.41	16.69	20.55	5137.5	0.267
ES	38	17.57	14.09	15.83	3959.3	0.300
HN	70	19.72	14.09	17.31	4577.0	0.200
HS	139	15.37	15.14	15.26	3814.3	0.224
LA	39	8.44	10.32	9.38	2343.8	0.497
SC	11	NA	12.46	12.46	3115.0	0.067
SH	44	13.57	17.89	15.73	3930.0	0.356
ST	50	18.36	18.08	18.22	4555.0	0.214
TE	35	NA	17.35	17.35	4335.0	0.357
WI	49	12.18	14.93	13.55	3387.5	0.254
WS	37	NA	10.57	10.57	2642.5	0.310*

^1^2005

^2^2007

*rock turn surveys (see Methods)

We classified habitat by the dominant vegetation type present at each collection site across the island and following the Holland Code classification system ([38]; S2 Fig). The Maritime Succulent Scrub (MSS) habitat was split into two subcategories based on whether the structurally dominant plant species was boxthorn (*Lycium sp*.; MSS-Ly, N = 5 sites) or cactus (*Opuntia* or *Cylidropuntia sp*.; MSS-Op, N = 4 sites). The grassland habitats (N = 3 sites) were any location with more than 75% grass (*Stipa sp*.) cover, even if the landscapes were a composite that included some minor component of MSS or other habitat (Fig 1b).

### Primer design and locus amplification

To identify variable microsatellite loci with reliable amplification within *X*. *riversiana*, we a) screened primer sets designed for microsatellites in the congener *X*. *vigilis* [39] and b) created a *de novo* enriched library of microsatellite loci using DNA extracted from four *X*. *riversiana* from San Clemente Island. DNA was extracted from tail tips stored in ethanol using Qiagen DNEasy kits, and four individuals were pooled for library construction. We then screened this genomic DNA for di- and tetranucleotide (CA and AAAG) repeats following the protocol in [40]. From this screen, we sequenced 96 prospective clones and identified 60 unique microsatellite motifs, including the motifs screened for and motifs we opportunistically identified in the cloned sequences. (GenBank accession numbers KT696132-KT696166). We designed primers using Primer3 ([41]; S1 Table). Six of these presumptive loci, as well as two of the loci from *X*. *vigilis* (N = 8 total loci), amplified reliably. We simultaneously amplified these loci with Qiagen Multiplex PCR Kits according to manufacturer's instructions (final reaction volume was 10μl), with at least 5 loci amplifying in 516 of the samples (Dryad repository DOI: doi:10.5061/dryad.6c7p5).

Since we had samples from multiple years in several sites, we ensured that each genotype was unique. We found no duplicate genotypes in our sample. We tested for differentiation between year-cohorts in the populations that had been sampled in multiple years (S2 Table). We used a per-locus exact G test implemented in Genepop with 100 batches of 100 iterations each, with 1000 dememorization steps [42, 43]. Loci were largely undifferentiated between years, with the exception of three populations (LA, SC, and ST) that had less than ten individuals sampled in 2005, and HN and HS, which showed differentiation in one and four loci, respectively. With this caveat in mind, we combined genotypes across years for our remaining analyses. To assess the quality of our markers, we calculated the number of alleles per locus, the observed and expected heterozygosity, polymorphism information content, and random match probability for each locus ([44]; S1 Table). We use the last two metrics as a proxy for the reliability of our dataset for distinguishing population-level patterns of differentiation.

### Next generation sequencing

In a brief exploration of potential marker skew, we genotyped three *X*. *riversiana*, one each from high, medium, and low elevations, using a double-digest RADseq approach followed by sequencing on an Illumina HiSeq platform [45]. Using the program pyRAD [46], we clustered the resulting sequences at 85% similarity across the three individuals, and retained the sequences that had at least one polymorphism, resulting in 3136 informative loci. For comparative purposes, we include seven individuals from the congeneric *Xantusia vigilis* that were sampled from populations in mainland central California (data not shown).

### Locus linkage and disequilibrium

To test for sex linkage, we reduced the data to the 130 individuals for which sex was positively known (N = 83 females, 47 males). We performed a chi-square analysis on the allele frequencies at each locus, and found no significant sex linkage. Three loci (XrivB1, XrivG2, and XrivY3) were homozygous for all individuals whose sex had been recorded and were not included in this analysis.

To test for deviations from Hardy-Weinberg equilibrium, we performed an exact test in Arlequin [47] using a 100,000 step Markov Chain with 1,000 burn in steps [48]. The test returns a *P*-value that indicates the two-tailed likelihood of observed heterozygosity in each locus and population. We then used Microchecker [49] to identify null alleles and their frequency, large allele dropout, and stutter by simulating 1000 randomizations to find the expected numbers of heterozygotes in each sampling location, assuming Hardy-Weinberg equilibrium. We set the confidence interval to 95%, Bonferroni corrected.

To test for linkage disequilibrium, we built contingency tables of observed allele frequencies at each pair of loci in Arlequin. We then permuted the genotypes and used a 1000 step Markov chain to explore the contingency table space for each pair and obtain the probabilities of the observed contingency tables.

### Genetic diversity, gene flow, and population structure

To estimate structure among collection locations across the island, we calculated average genetic diversity and location-specific F_ST_ and F_IS_ in Arlequin. We identified private alleles using Genalex [50] for each collection location. We calculated an indirect measure of gene flow using the formula F_ST_ = 1/(4N_m_+1), rearranged to solve for N_m_, the number of migrants per time step [51]. We used BayesAss to find an alternate measure for asymmetric migration rates between pairs of populations [52].

As a further test of population structure, we used the program Tess [53] to identify genetic demes and the proportion of each individual’s genome that belongs to those demes. For any individuals for which we did not have unique collection locations, we generated unique coordinates sampled from a 50 by 50 meter square centered on the coordinates for the entire collection site. For individuals caught in the same pitfall trap, we perturbed the collection location of all but one individual by one meter in a randomly chosen cardinal direction.

We computed pairwise geographic distances using the Euclidean option due to many of our samples being in close proximity. We began our runs with the no-admixture model with the spatial interaction parameter set to zero to mimic the Structure algorithm. Visual assessment of this analysis indicated that the optimal number of clusters was around 5, so we concentrated on K_max_ values between 3 and 7. We then implemented the CAR admixture model. DIC values for short runs of this model indicated that the optimal K_max_ was between 3 and 5. We refined this estimate by looking at the proportion of the available genomes that were assigned to each cluster. For K_max_ of 4, one cluster had less than 5 percent of the available genomes assigned to it, indicating that it was a dummy cluster that had not completely emptied after 40,000 iterations. The same was true for two clusters for K_max_ of 5. Thus, we concentrated our analysis on the runs with a K_max_ of 3. We repeated the analysis with several variations of subsamples versions of the two populations for which we had more than 60 individuals, and we found the results to be very robust to all permutations.

### Effective population size

Island populations can show much lower effective population sizes relative to census size [6, 7]. To assess the difference between effective and census size estimates, we used coalescent simulations in Lamarc to determine effective population size at each collection location [54]. For locations with more than 15 individuals sampled in a year, we randomly subsampled two sets of 10–15 genotypes. We calculated the parameter theta (4N_e_μ) five times from each data set and averaged those calculations. Finally, we generated the mean of the means of the two subsamples of individuals. We find a relative measure of population size by standardizing our theta values by our lowest reconstructed size. We used the standard microsatellite mutation rate of 0.001 mutations per individual per generation to calculated N_e_ for each collection location [55]. We compared the results to demographic capture rate (lizard/trap/day) data [56] for the locations at which those data are available, including an estimation of capture rates at rock turn sites calculated from an average rate of two rocks flipped and replaced per minute, which includes lizard handling time (lizards/hours sampled/daily flip rate; Table 1).

To calculate the effective population size for all night lizards on San Clemente, we used the ‘pegas’ package [57] in R ver. 2.12.1 (R Core Development Team 2010) to find the value for theta calculated across all individuals and all loci. We calculated three values for theta [58, 59], and used the formula *θ* = 4N_e_μ to calculate effective population size. We used a value of 0.001 for the microsatellite mutation rate [55] and averaged across loci to find the mean and standard deviation for the effective population size.

We used the library “pegas” in R to calculate theta based on heterozygosity for each SNP from our ddRAD dataset [57]. We calculated N_e_ using the standard equation *θ* = 4N_e_μ. We set μto the standard estimate for sequence mutation rate, 2.5 x 10^−8^ [60]. We contrasted this estimate with two mainland effective population size estimates. The first was from *Xantusia vigilis* from the Panoche Hills in the central part of mainland California. We also used *X*. *vigilis* from the Cuyama Hills. We repeated the analysis with one *X*. *vigilis* drawn from Pinnacles National Park, the Panoche Hills, and the Cuyama Hills.

### Population bottlenecks

There are have been two major reductions in habitat availability in the recent history of San Clemente. The first was the forty percent reduction in size the island suffered as sea levels rose after the LGM, which would have primarily effected the low-lying night lizard populations. The second was the century-long habitat degradation by goats, which was more severe on the higher elevation areas. To determine the relative impacts of these two events, we used the Garza-Williamson (GW) index to identify any reductions in effective population size in each location [19]. We calculated the statistic for each locus in Arlequin, and report the mean and standard deviation of those calculations. The GW index is vulnerable to inaccurate assumptions about the mutation model. As we do not know the mutation rate of our loci, we use a comparative approach in our analysis. We regard populations with relatively low GW indices as having experienced greater relative declines than those with higher indices. If a population is bottlenecked and then recovers, simulations show that the GW index will return to near its pre-bottleneck levels within 400 generations [61]. Thus, any detected perturbations should indicate a fairly recent demographic event. If vegetation loss has caused a major bottleneck in grass-living populations, then this statistic would be lower in these populations than those living in Maritime Succulent Scrub (MSS) habitats.

### Spatial genetic structure

We used a discriminant analysis of principle components implemented in the R package ‘adegenet’ to assess spatial genetic structures [62]. We calculated the coordinates of each individual on the first two PC axes, and from these found the centroid for each collection location.

We used the ‘Raster’ [63] package in R to extract the elevations of our collection locations from the DEM, and assigned each collection location a habitat type based on observation during collection (Fig 1B). We performed an ANOVA on the elevations in R with the Tess deme identity of each location as a three level factor. We investigated the correlation between deme identity and potential explanatory variables by using a Generalized Additive Model (GAM) implemented in the R package ‘gam’ [64]. For each deme, we performed a GAM with just elevation, just habitat, and elevation + habitat as the explanatory variables. We calculated relative AIC values for each model calculated for each deme. The model was unable to definitively distinguish between elevation and elevation + habitat for the medium and high demes, and selected elevation as the best explanatory variable for the low deme. We therefore used elevation to extrapolate the probable deme identity of populations below the current water line. We used a bathymetry raster publicly available from the NOAA National Geophysical Data Center [65], and used the latitude, longitude and elevation information for each raster cell to provide the independent variables for the model. The dependent variable was the percentage of identity in the Tess-identified demes at each sampling location. We did a separate GAM for each of the demes.

In order to assess the correlation between genetic distance and geographic features, we used Mantel tests implemented in IBDWS [66]. To generate our pairwise geographic resistance matrices, we used the program Circuitscape [67]. The rows and columns of the matrices were randomized and the correlation statistic was recalculated for each randomization and compared to the original statistic to generate a p-value for the significance of the correlation. We used the pairwise F_ST_ matrix from Arlequin for genetic distance. Circuitscape models a habitat raster as grid nodes, each with a specified resistance. The model calculates the total resistance between each pair of population locations.

We obtained a 7.5 minute digital elevation model (DEM) map from webGIS [68–73]. We specified habitat rasters by calculating slope from a digital elevation model raster using the ‘raster’ package in R [63]. We calculated pairwise resistances between collection locations for the slope raster, and for a raster in which every cell was set to the mean value, such that it had the same total resistance but did not include any slope information. This raster served as a test for strict isolation by distance, rather than isolation by resistance.

## Results

### Locus behavior, genetic diversity, and population structure

None of the five loci with variation among adults were sex linked (*P*-values between 0.115 and 0.630). The number of alleles ranged from four to 32, with a total of 93 unique alleles. Observed heterozygosity was less than expected heterozygosity for all loci except XrivR1. Random match probability, roughly a measure of the probability of any two individuals in the population having the same multilocus genotype, was 4.37x10^-6^. We calculated polymorphic information content and random match probability for each locus (S1 Table). We found an excess of homozygotes in three populations for locus B1, two populations for G1, and one population each for G2 and Y3, but no other deviations were detected. Two sites had one or more loci that were out of Hardy-Weinberg equilibrium, and every locus was out of equilibrium for at least one collection site (S3 Table). However, there were no consistent trends in the lack of equilibrium across loci and populations, so we retained all loci for our further analysis. We found no, or very minimal, evidence of any linkage disequilibrium in most sites. However, one site on the low-lying western side of the island (HN) had three pairs of loci in linkage disequilibrium (S4 Table).

Across all collection sites, the average proportion of polymorphic loci was 6.33/8 loci, with the minimum (two sites) having five polymorphic loci, and the maximum (two sites) having eight polymorphic loci (S5 Table). The mean number of alleles per site was 6.17 ± 5.05 (S6 Table). Average gene diversity (P) was not significantly different for any sampling site, with all locations showing a value near 0.4 (± 0.2). F_IS_ values were also between -0.11 and 0.012, while location-specific F_ST_ values were between 0.098 and 0.1. The majority of the population-specific private alleles occurred in HN and HS (S6 Table). Pairwise F_ST_ values ranged from 0.1575 between BO and LA, and 0.0047 between HN and HS (S8 Table).

**Fig 2:**
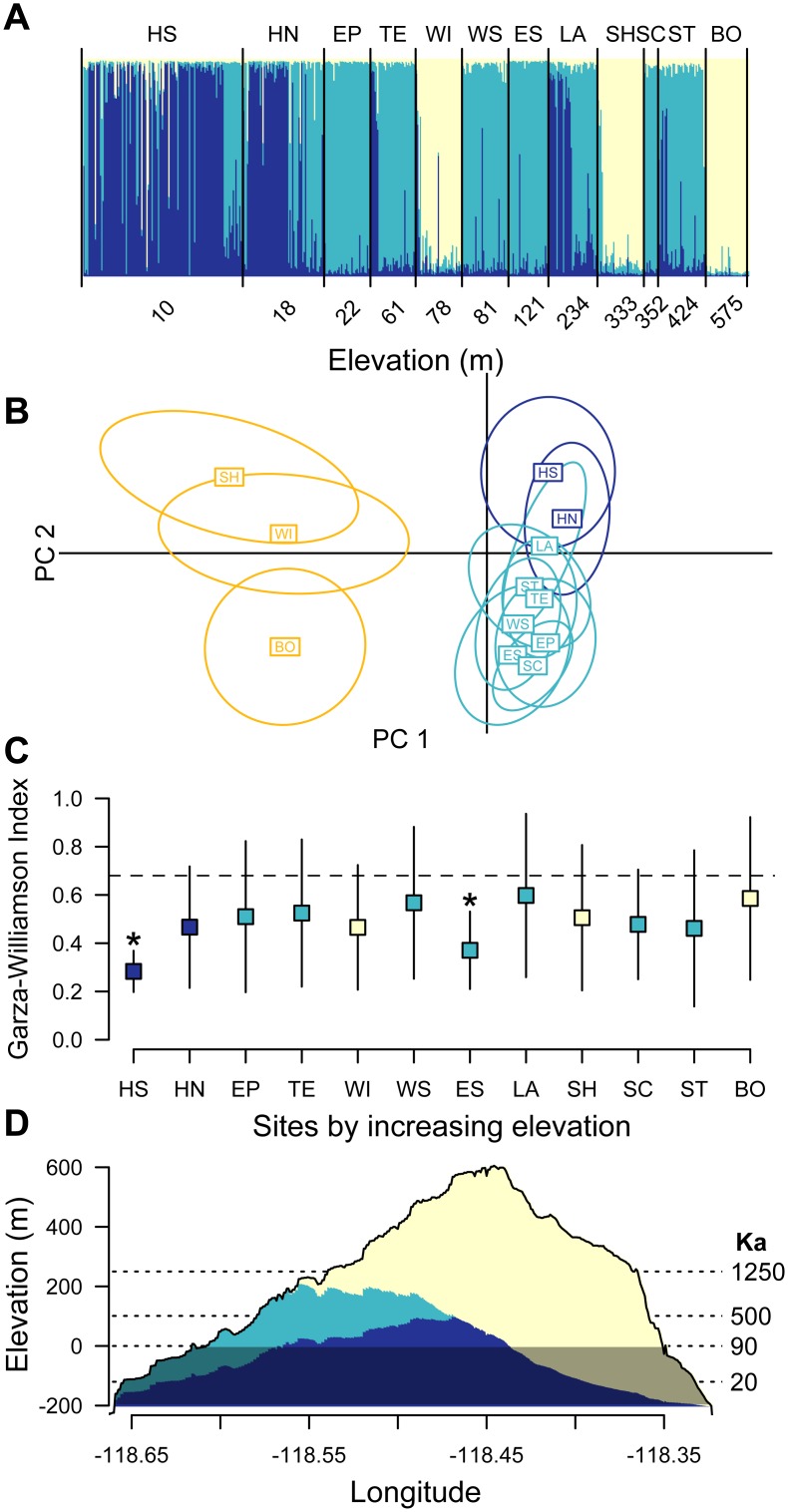
*Xantusia riversiana* genetic demes follow elevation on San Clemente Island. a Percentages of deme identity found for each individual by collection location generally correspond to elevation, with a low (dark blue), mid (light blue), and high elevation (cream) deme. b Discriminant analysis of principle components concordantly recovers three clusters. Locations are color-coded according to the Tess deme that had the highest representation at that location. c Mean ±1 s.e.m. of the Garza-Williamson Index for each collection location shows two sites with evidence of historical bottleneck (asterisks). d Elevational profile plot of the highest point on San Clemente Island at each longitude, colored by deme representation from the GAM prediction. Gray shading denotes area currently below sea level, and dashed lines show historical sea levels

We found the highest levels of migration among the populations in the central third of the island, ranging from 15–53 migrants per generation (Fig 1c). All other pairwise populations exchange less than ten migrants per generation (S7 Table). These levels of migration must be interpreted in context of the effective population sizes of the populations. BayesAss showed the highest migration values from ES to EP, from LA and HN to HS, from SC and TE to ST, and WI to WS (S9 Table). All of these pairs of populations are geographically close. The most distant pair is LA to HS, but this result is mirrored by the Tess analysis (Fig 2a).

The Tess analysis recovered three regional demes (Fig 2a). One deme is made up of two collection locations at the southern end of the island combined with the northern most collection location, which is 23 km from these southern localities. A second deme covers the intervening central populations, while a third is concentrated around the lowest elevation sites of HN and HS, with considerable representation in the LA and TE sites. For convenience, we use the terms low, medium, and high to describe the demes, based on the relative elevations of the majority of the populations.

We independently estimated the deme affiliation of each collection location using Principle Components Analysis. We again recovered three groups, mainly differentiated along PC1, with membership generally concordant with the Tess analysis: the southern sites of BO and SH, the low elevation HN and HS sites, and then the remaining sites. The main difference between the two analyses is that WI does not group with BO and SH. PC2 loosely differentiates LA, WI and WS, although their convex hulls overlap other populations (Fig 2b).

### Effective population size and genetic bottlenecks

Standardized population sizes ranged from 1 to 2.2, with generally larger theta values in the HS, HN, and EP collection locations. Using a mutation rate of 0.001 mutations per individual per generation, we calculated estimates for effective population size for each collection location, which ranged from ~2300–5100 individuals per site (Table 1). We also found that these site-specific effective population sizes were not correlated with capture rates (F_1,10_ = 1.47, *P* = 0.254), even when rock turn sites were excluded (F_1,8_ = 0.64, *P* = 0.447). For the entire island, we found estimates of effective population size to range from 42,083 (±79,060) to 62,109 (±106,308) individuals, depending on the method used to calculate theta.

Two sites on the western MSS-rich side of the island (HS and ES) show relatively low Garza-Williamson index compared to the other sites, indicating that they have undergone proportionally greater population reductions in recent history than the other sampled locations (Fig 2c). Their upper standard error bars fall below the 0.68 cutoff that Garza and Williamson found to indicate a history of a bottleneck in empirical and simulated populations.

The ddRAD dataset had 1046 polymorphic loci across one individual each from the high elevation, middle elevation, and low elevation demes, and gave us an effective population size of 3,178,936. We combined sets of three *Xantusia vigilis* from a total of seven samples taken across central California. The effective population sizes ranged from 3,235,742 to 3,836,402 depending on the grouping we used.

### Spatial genetic structure

We found that the first two principle component axes isolated the “high” demes from the “low” and “medium” demes (Fig 2b). Both the isolation by slope (*P* = 0.0049) and the isolation by distance (*P* = 0.0094) rasters showed a significant relationship with genetic distance. Reduced Major Axis regression showed a tighter correlation and higher slope (slope = 0.7402, R^2^ = 0.218) for the isolation by slope than the isolation by distance test (slope = 0.4402, R^2^ = 0.183). The output slope raster had values between 0 and 1.0037, with a mean value of 0.203 (Fig 1c shows slopes). Overall, we found a better, although still weak, correlation between genetic deme and elevation (t = -2.108, df = 10, *P* = 0.061) than between deme and habitat (χ^2^ = 3.45, df = 4, *P* = 0.486). The effect of elevation was strongest on the mid-elevation deme (F_1,10_ = 5.001, *P* = 0.049), although not significant for the high (F_1,10_ = 2.05, *P* = 0.182) or low demes (*F*_1,10_ = 2.07, *P* = 0.181). The GAM models for the high deme showed relative AIC’s of 0.13, 0.01, and one for the only elevation, only habitat, and habitat + elevation models, respectively. The mid-elevation deme showed relative AIC scores of 0.25, 0.07, and one for the three models, and the high-elevation deme showed relative scores of one, 0.29, and 0.14 for the three models. Using the GAM models, the projected deme affiliation across the island suggests that most of the island’s area has lizards from the high elevation deme, with the low elevation (and high genetic diversity) deme restricted to only a small fraction of the total above-water island area (Fig 2d).

## Discussion

We found no evidence of recent bottlenecks in any of the populations, regardless of the degree of degradation in the local habitat. We found evidence of an older bottleneck detected by the M-ratio tests in two low-elevation populations. Goats were introduced 150 years ago, which, assuming a lizard generation time of at least 5 years (estimated lifespan of 13+ years; [27]), represents no more than 30 generations for *X*. *riversiana* [32]. Bottlenecks severe enough to results in measurable reduction in population genetic diversity generally must continue for at least 50 generations, given a reasonable population size [61]. Our results indicate that the eradication of the goats occurred quickly enough after their introduction that genetic diversity was maintained in even the most heavily impacted populations. This finding suggests that ongoing management for re-vegetation should have positive impacts on the island night lizard. It also indicates that other island herbivore removal projects could be useful applications of conservation effort, since similar species may also have maintained genetically healthy populations despite long-term habitat degradation. However, our relatively low number of microsatellite loci and low allelic richness of these loci mean that our results should be interpreted with caution. Further genetic work should be conducted on the night lizards to confirm our recommendations.

### Genetic diversity of *Xantusia riversiana*

We found that pairwise genetic distance between collection locations increased with the pairwise geographic distance and the degree of slope between two populations, which could account for the roughly elevationally stratified demes. In practice, it indicates that low elevation populations should not be counted on to provide natural demographic or genetic “rescues” of higher elevation populations in this low-vagility species [74]. Currently, the majority of high quality MSS habitat on San Clemente Island is at low elevations, and higher elevations are still degraded from the effects of past herbivory by introduced grazers, although these habitats have continued to recover post-removal of goats. The high elevation locations we sampled had lower capture rates than the low elevation populations, indicating potential demographic fragility in those areas, despite their lack of evidence for genetic bottlenecks (Table 1). The low elevation deme also contained genetic variation of conservation priority, with a relatively high proportion of private alleles and limited distribution across the island. Some low elevation populations also had a signature of a past genetic bottleneck, indicating that the deme had undergone a large and likely long-term reduction in population size in the past. This finding is consistent with the conjecture that night lizards belonging to the low deme once occupied the area that was exposed in the Pleistocene, and the deme underwent dramatic reduction as the area became submerged.

An indirect comparison of these genetic variation levels to the mainland congener *X*. *vigilis* (see [37]) suggests *X*. *riversiana* on San Clemente Island has about half the variation of one population of *X*. *vigilis* across multiple metrics (proportion of polymorphic loci, observed heterozygosity, number of unique alleles per locus). Reduced genetic variation is a classic characteristic of island fauna, and special care should be taken to preserve the diversity that does exists across the island. However, we suspect that our overall estimate of island-wide effective population size (mean N_e_ = 50,000) may be significantly lower than the true N_e_, especially considering the estimated demographic census size (N) of approximately 21 million individuals [55]. Analysis of RADSeq loci using one individual from each of the three demes on San Clemente Island yielded a N_e_ of 3.18 million, which is far closer to the expected N_e_ in a lizard system given the large size estimated for N. Further study of this species should be undertaken to better understand the true absolute and effective population sizes. The differences in effective population size estimates between the sequence data and microsatellite data may have several causes. We may not have enough microsatellite loci to adequately describe the effective population size, while the large number of loci in our RADseq data more accurately capture actual effective population size. Identifying the primary causes of the differences in estimates between the microsatellites and sequence data will take future research on the reaction of different genetic marker types to various demographic scenarios.

As *Xantusia riversiana* also inhabits two other Channel Islands (Fig 1a), the greatest outstanding questions about their genetics are 1) what is the genetic diversity on the other two islands, and 2) how long ago did the populations split? The best available genetic evidence (allozyme and karyotyping data for many individuals [75] and Sanger sequencing data for a few samples from each island [23]) suggests that these three island populations are each quite distinct and have been separated without gene flow for at least 500,000 years, with the greatest standing variation in both allelic diversity and color pattern of the three populations being found on San Nicolas Island. A thorough investigation and comparison of genetic diversity across islands will be essential to management of the species as an integrated unit.

### Effects of previous conservation action: goat removal

The conservation concern that prompted *Xantusia riversiana*’s listing as federally threatened was habitat destruction due to invasive herbivores, farming, and invasive plants [75]. The goats have since been eradicated, and native flora is rebounding under the management of the U.S. Navy through targeted re-vegetation efforts, natural recovery, and invasive species control [32, 33]. We found no greater evidence of genetic bottlenecking in areas where the vegetation was heavily degraded due to past goat herbivory than in areas in which it remained more pristine. Our results indicate that island populations may be genetically robust to some types of multi-decade habitat degradation. In populations in which this is the case, invasive species removal can be a worthwhile conservation measure.

### Future conservation focus

Since the removal of feral goats, investment into native plant restoration, feral cat management, erosion control, and the creation of Island Night Lizard Management Areas (INLMA) across key MSS habitat have helped ensure population stability of *Xantusia* on San Clemente Island. This study adds key information about the main dispersal corridors across the middle of the island and demonstrating the importance of elevation in predicting genetic variation across sea terraces, which should help with future land management decisions.

Climate change models indicate that, in the absence of a worldwide reduction in carbon output by 2050, we should be prepared for sea level rise of at least 0.3 meters by 2100 [76]. Although the population currently shows signs of demographic health, the sea-level HS/HN sites are at more risk than the other areas. The HS/HN sites also contain a disproportionate number of private alleles, indicating that they contain important genetic resources for the species. As slopes are barriers to dispersal, and these collection locations are surrounded by sea terraces (Fig 1c), the natural dispersal process of *X*. *riversiana* may be inadequate to offset habitat contraction. For example, sea levels rose continually between 13,500 years ago and 2,000 years ago without homogenizing the genetic differences between these sites and some of their nearest neighbors, suggesting very low up-slope dispersal. Depending on the trajectory of sea level rise, future conservation efforts may need to focus on safeguarding this population. Similar species may follow this pattern, indicating that managers should determine whether their species have clusters of genetic diversity that could be at risk due to sea-level rise.

## Supporting Information

S1 Fig *Xantusia riversiana reticulata*, the Island Night Lizard, from San Clemente Island, California, USA. (JPG)

S2 Fig Major habitat types on San Clemente Island. a Maritime Succulent Scrub, *Opuntia* phase (MSS-Op). b Maritime Succulent Scrub, *Lycium* phase (MSS-Ly). c Grassland (primarily *Stipa sp*.), with secondary invasion of shrubs (*Heteromeles sp*.) post-removal of feral goats.
(PNG)

S1 Table Locus characteristics. Characteristics of eight multiplexed microsatellite loci in *Xantusia riversiana reticulata* (*N* = 516 individuals). Sequences for 54 additional candidate clones are available through GenBank (KT696132-KT696166). Note abbreviations for number of alleles (N) and observed (*H*_*o*_), expected (*H*_*e*_) heterozygosities, polymorphism information content (PIC), and random match probability (RMP). All loci were out of Hardy-Weinberg equilibrium when analyzed island-wide, but not by population (S3 Table).
(DOCX)

S2 Table Year-cohort differentiation. *P-*values of G tests of genetic differentiation between year-cohorts for populations sampled in multiple years. A dash denotes monomorphic locus-population combinations for which differentation cannot be assessed. The final two columns show the whole-population Chi^2^ statistic, and the whole-population *P*-value.
(DOCX)

S3 Table Per locus and population Hardy-Weinberg equilibrium. *P*-values for tests of deviation from Hardy-Weinberg equilibrium (HWE) per locus (columns) and collection site (rows). A dash denotes monomorphic locus-population combinations for which HWE cannot be assessed. Bold lettering denotes significant deviation after Bonferroni correction (*N* = 6).
(DOCX)

S4 Table Loci in linkage disequilibrium per site. Linkage disequilibrium between pairs of loci by collection site. Bold lettering denotes significant linkage after Bonferroni correction (*N* = 7).
(DOCX)

S5 Table Per locus and population allele counts. Allele counts and summary statistics for each locus (rows) by collection site (columns).
(DOCX)

S6 Table Private alleles (*N* = 12) by collection location. Sites in bold (10 of 12) are all within 5km of each other on the low-lying western side of the island characterized by high quality MSS habitat (see Fig 1b).
(DOCX)

S7 Table Pairwise numbers of migrants. Number of migrants (N_m_) per generation between pairs of collection sites. Migration rates higher than 5 are in bold.
(DOCX)

S8 Table Pairwise F_ST_ between all pairs of populations. (DOCX)

S9 Table Pairwise BayesAss measurements between all pairs of populations. (DOCX)
