# Supplementary figures and images for: Historical Environment Is Reflected in Modern Population Genetics and Biogeography of an Island Endemic Lizard (Xantusia riversiana reticulata)
Source: PLoS One. 2016 Nov 9;11(11):e0163738. doi: 10.1371/journal.pone.0163738 (PMC5102444; doi:10.1371/journal.pone.0163738)

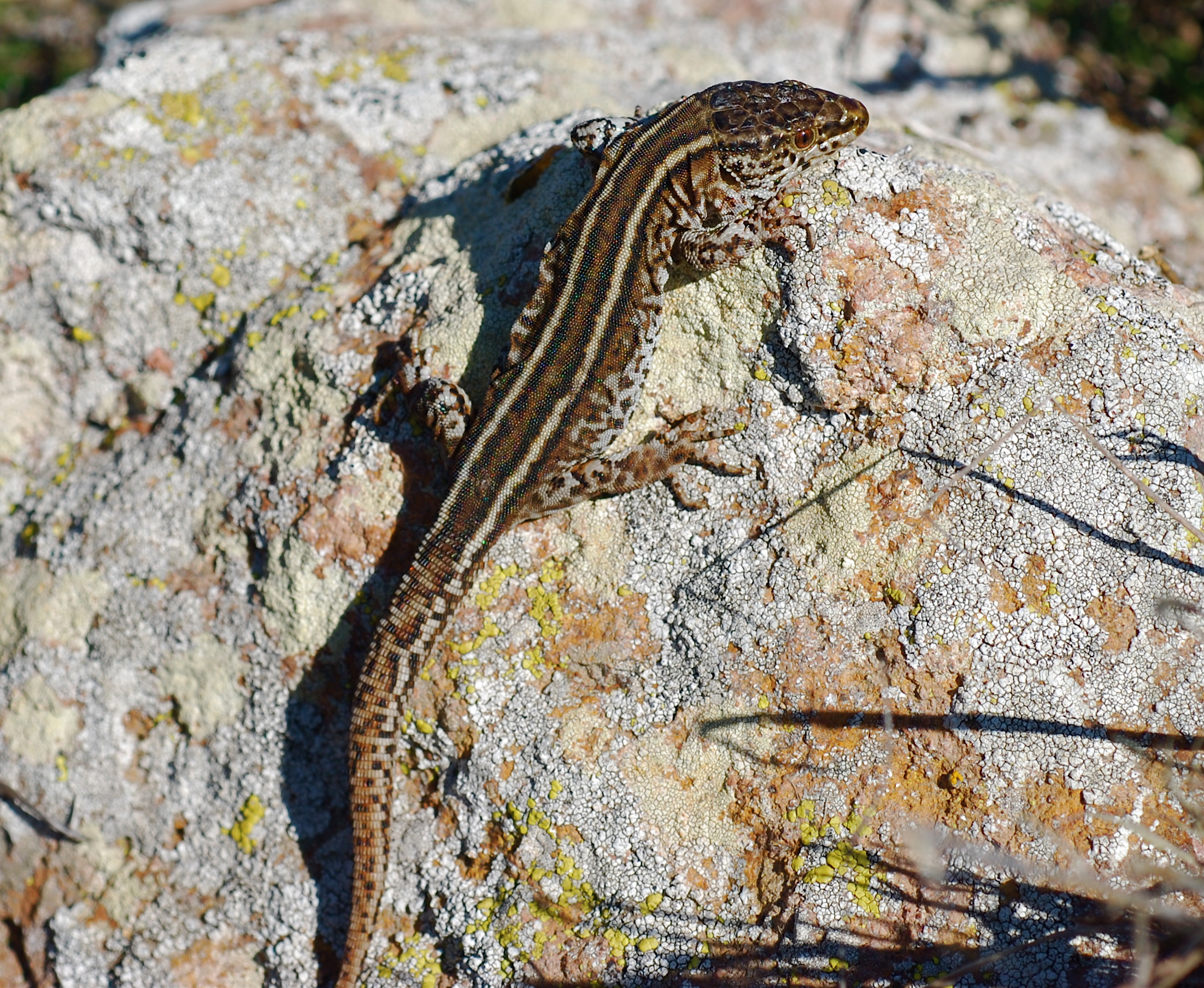

Supplement: S1 Fig — (JPG) [file pone.0163738.s001.jpg]

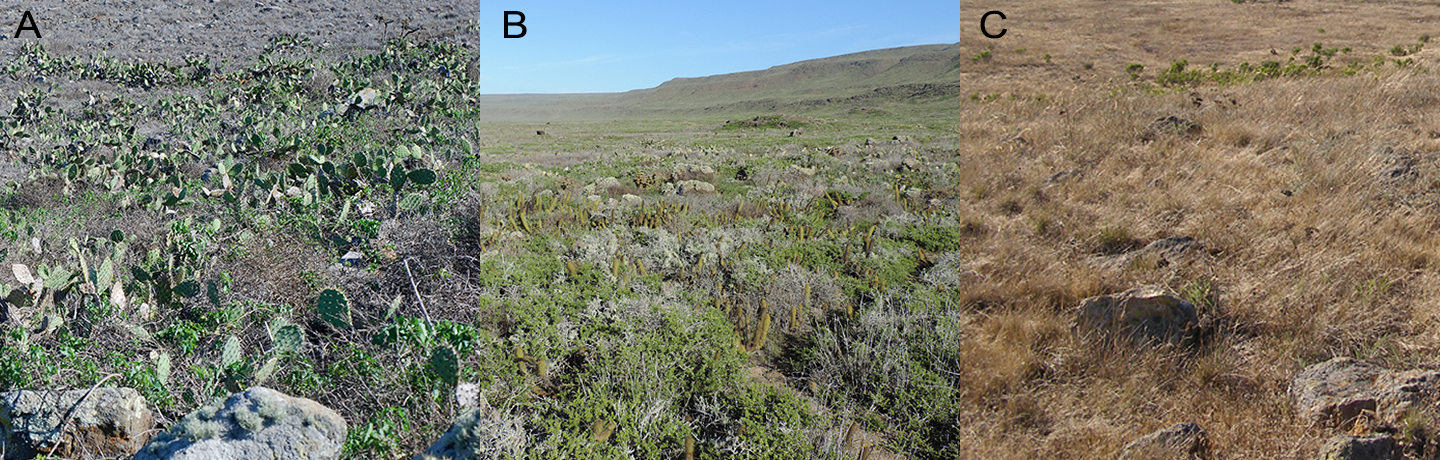

Supplement: S2 Fig — a Maritime Succulent Scrub, Opuntia phase (MSS-Op). b Maritime Succulent Scrub, Lycium phase (MSS-Ly). c Grassland (primarily Stipa sp.), with secondary invasion of shrubs (Heteromeles sp.) post-removal of feral goats. (PNG) [file pone.0163738.s002.png]
