# Supplementary material for: Historical Environment Is Reflected in Modern Population Genetics and Biogeography of an Island Endemic Lizard (Xantusia riversiana reticulata)
Source: PLoS One. 2016 Nov 9;11(11):e0163738. doi: 10.1371/journal.pone.0163738 (PMC5102444; doi:10.1371/journal.pone.0163738)
Supplement: S1 Table — Characteristics of eight multiplexed microsatellite loci in Xantusia riversiana reticulata (N = 516 individuals). Sequences for 54 additional candidate clones are available through GenBank (KT696132-KT696166). Note abbreviations for number of alleles (N) and observed (Ho), expected (He) heterozygosities, polymorphism information content (PIC), and random match probability (RMP). All loci were out of Hardy-Weinberg equilibrium when analyzed island-wide, but not by population (S3 Table). (DOCX) [file pone.0163738.s003.docx]

S1 Table. Locus characteristics. Characteristics of eight multiplexed microsatellite loci in *Xantusia riversiana reticulata* (*N*=516 individuals). Sequences for 54 additional candidate clones are available through GenBank (KT696132-KT696166). Note abbreviations for number of alleles (N) and observed (*H_o_*), expected (*H_e_*) heterozygosities, polymorphism information content (PIC), and random match probability (RMP). All loci were out of Hardy-Weinberg equilibrium when analyzed island-wide, but not by population (Table S3).

| **Locus** | **Repeat**  **Motif** | **Primer Sequence (5**′**-3**′**)**  **(F=*dye*-forward, R=reverse)** | **Range**  **(bp)** | **N** | ***H_o_*** | ***H_e_*** | | **PIC** | **RMP** | | **GenBank**  **Accession #** | |  |
| --- | --- | --- | --- | --- | --- | --- | --- | --- | --- | --- | --- | --- | --- |
| XrivB1 | CTT | F: *FAM*-TCGCATCCACCTACACAAGC  R: GGTTTGGTGTGCTGCCTAGT | 125-132 | 4 | 0.000 | 0.019 | 0.019 | | | 0.981 | | KT833329 | |
| XvGLA | AAAG | F: *FAM*-TTGCCTGTCCCAAAAGTCTC  R: CCTGACTGGAAGGAGCTCAG | 260-346 | 32 | 0.888 | 0.931 | 0.931 | | | 0.009 | | FJ197164 | |
| XrivG2 | TG | F: *VIC*-ACACTCTGCTCCCCTTCAGA  R: GCCCAAGGTTACCCAGTGAG | 130-134 | 5 | 0.008 | 0.025 | 0.025 | | | 0.969 | | KT696148 | |
| XrivG1 | GT | F: *VIC*-AAGCTTCGCATCCAGCAGTT  R: CCCTTTCATCCGTTGCCAGA | 182-220 | 14 | 0.655 | 0.748 | 0.748 | | | 0.116 | | KT833330 | |
| XvCHEL | AAAG | F: *VIC*-ATGTTTTCCTGTCCCAAAGG  R: GGCAAGCTATCCTCTGCTTG | 250-290 | 14 | 0.727 | 0.750 | 0.750 | | | 0.079 | | FJ197163 | |
| XrivY3 | CTT | F: *NED*-AGCTTCGAGCCGATCTTGAG  R: AGCAGGAACATCACTCCACG | 263-271 | 7 | 0.025 | 0.029 | 0.029 | | | 0.947 | | KT833332 | |
| XrivR1 | ACC | F: *PET*-TCCCATGCACAGCAAAAAGC  R: CCTTGCCCTCCAAGAAGGTT | 142-176 | 10 | 0.558 | 0.485 | 0.484 | | | 0.254 | | KT833331 | |
| XrivR2 | CAA | F: *PET*-AAGACAGCCGCCAAATCCTT  R: GAATGGTGCGTAGACGGTGA | 224-234 | 7 | 0.393 | 0.609 | 0.609 | | | 0.231 | | KT696134 | |
