# Supplementary material for: Historical Environment Is Reflected in Modern Population Genetics and Biogeography of an Island Endemic Lizard (Xantusia riversiana reticulata)
Source: PLoS One. 2016 Nov 9;11(11):e0163738. doi: 10.1371/journal.pone.0163738 (PMC5102444; doi:10.1371/journal.pone.0163738)
Supplement: S2 Table — P-values of G tests of genetic differentiation between year-cohorts for populations sampled in multiple years. A dash denotes monomorphic locus-population combinations for which differentation cannot be assessed. The final two columns show the whole-population Chi2 statistic, and the whole-population P-value. (DOCX) [file pone.0163738.s004.docx]

S2 Table. Year-cohort differentiation. *P-*values of G tests of genetic differentiation between year-cohorts for populations sampled in multiple years. A dash denotes monomorphic locus-population combinations for which differentation cannot be assessed. The final two columns show the whole-population Chi^2^ statistic, and the whole-population *P*-value.

|  | **Xriv**  **B1** | **Xv**  **GLA** | **Xriv**  **G2** | **Xriv**  **G1** | **Xv**  **CHEL** | **Xriv**  **Y3** | **Xriv**  **R1** | **Xriv**  **R2** | **Chi^2^** | **P-val** |
| --- | --- | --- | --- | --- | --- | --- | --- | --- | --- | --- |
| **EP** | - | 0.664 | - | 0.084 | 0.910 | 0.362 | 0.888 | 1.000 | 8.24 (12) | 0.766 |
| **ES** | 0.646 | 0.772 | 0.646 | 0.622 | 0.520 | 1.000 | 0.925 | 0.348 | 6.786 (16) | 0.977 |
| **HN** | 0.201 | 0.378 | 0.194 | 0.001 | 0.000 | - | 0.000 | 0.000 | Inf  (14) | 0.000 |
| **HS** | 0.581 | 0.151 | 0.789 | 0.809 | 0.163 | 0582 | 0.391 | 0.000 | 29.83 (16) | 0.019 |
| **LA** | - | 0.429 | - | 0.451 | 0.339 | - | 0.000 | 0.000 | Inf  (10) | 0.000 |
| **SC** | 0.000 | 0.000 | 0.000 | 0.000 | 0.000 | 0.000 | 0.000 | 0.000 | Inf  (16) | 0.000 |
| **SH** | - | 0.266 | - | 0.235 | 0.850 | 0.855 | 0.396 | 0.612 | 9.01 (12) | 0.702 |
| **ST** | - | 0.262 | - | 0.001 | 0.000 | 1.000 | 0.024 | 0.004 | 49.91 (12) | 0.000 |
| **WI** | - | 0.687 | 1.000 | 0.393 | 0.617 | - | 0.391 | 0.266 | 8.11 (12) | 0.777 |
