# Supplementary material for: Historical Environment Is Reflected in Modern Population Genetics and Biogeography of an Island Endemic Lizard (Xantusia riversiana reticulata)
Source: PLoS One. 2016 Nov 9;11(11):e0163738. doi: 10.1371/journal.pone.0163738 (PMC5102444; doi:10.1371/journal.pone.0163738)
Supplement: S3 Table — P-values for tests of deviation from Hardy-Weinberg equilibrium (HWE) per locus (columns) and collection site (rows). A dash denotes monomorphic locus-population combinations for which HWE cannot be assessed. Bold lettering denotes significant deviation after Bonferroni correction (N = 6). (DOCX) [file pone.0163738.s005.docx]

S3 Table. Per locus and population Hardy-Weinberg equilibrium. *P*-values for tests of deviation from Hardy-Weinberg equilibrium (HWE) per locus (columns) and collection site (rows). A dash denotes monomorphic locus-population combinations for which HWE cannot be assessed. Bold lettering denotes significant deviation after Bonferroni correction (*N*=6).

|  | **Xriv**  **B1** | **Xv**  **GLA** | **Xriv**  **G2** | **Xriv**  **G1** | **Xv**  **CHEL** | **Xriv**  **Y3** | **Xriv**  **R1** | **Xriv**  **R2** |
| --- | --- | --- | --- | --- | --- | --- | --- | --- |
| **EP** | - | 0.210 | - | 0.080 | 0.381 | 1.000 | 1.000 | 0.381 |
| **ES** | 0.015 | 0.006 | 0.017 | 0.001 | 0.029 | 1.000 | 1.000 | 0.068 |
| **HN** | **0.000** | 0.004 | **0.000** | **0.000** | 0.102 | - | 0.256 | 0.001 |
| **HS** | 0.005 | 0.246 | 0.011 | **0.000** | 0.006 | 0.004 | **0.000** | **0.000** |
| **LA** | - | 0.433 | - | 0.019 | 0.591 | - | 0.50932 | 0.287 |
| **SC** | - | 0.505 | - | 0.061 | 0.929 | 1.000 | 1.000 | 1.000 |
| **SH** | - | 0.110 | - | 0.208 | 0.911 | 1.000 | 1.000 | 0.001 |
| **ST** | - | 0.118 | - | 0.701 | 0.784 | 1.000 | 0.952 | 0.002 |
| **TE** | - | 0.172 | - | 0.984 | 0.743 | 1.000 | 1.000 | 0.107 |
| **WI** | - | 0.619 | 1.000 | 0.217 | 0.195 | 1.000 | 0.657 | 0.008 |
| **WS** | - | 0.505 | - | 0.007 | 0.203 | 1.000 | 0.586 | 0.130 |
