# Supplementary material for: Historical Environment Is Reflected in Modern Population Genetics and Biogeography of an Island Endemic Lizard (Xantusia riversiana reticulata)
Source: PLoS One. 2016 Nov 9;11(11):e0163738. doi: 10.1371/journal.pone.0163738 (PMC5102444; doi:10.1371/journal.pone.0163738)
Supplement: S4 Table — Linkage disequilibrium between pairs of loci by collection site. Bold lettering denotes significant linkage after Bonferroni correction (N = 7). (DOCX) [file pone.0163738.s006.docx]

S4 Table. Loci in linkage disequilibrium per site. Linkage disequilibrium between pairs of loci by collection site. Bold lettering denotes significant linkage after Bonferroni correction (*N*=7).

| **Site** | **Locus 1** | **Locus 2** | ***P*-value** |
| --- | --- | --- | --- |
| BO | XvCHEL | XrivR2 | 0.033 |
| EP | XrivG1 | XrivR2 | 0.029 |
| ES | XvGLA | XrivG1 | 0.016 |
| ES | XrivB1 | XrivG2 | 0.033 |
| ES | XrivB1 | XrivY3 | 0.034 |
| ES | XrivG2 | XrivY3 | 0.035 |
| **HN** | **XrivB1** | **XrivG2** | **0.000** |
| **HN** | **XvCHEL** | **XrivR2** | **0.000** |
| **HN** | **XrivR1** | **XrivR2** | **0.000** |
| HN | XrivG1 | XrivR1 | 0.002 |
| HN | XvGLA | XrivR1 | 0.005 |
| HN | XvCHEL | XrivR1 | 0.010 |
| HN | XrivG1 | XrivR2 | 0.021 |
| HN | XrivG1 | vCHEL | 0.024 |
| **HS** | **XrivR1** | **XrivR2** | **0.000** |
| HS | XvCHEL | XrivR1 | 0.007 |
| HS | XrivB1 | XrivY3 | 0.007 |
| HS | XrivY3 | XrivR2 | 0.008 |
| HS | XrivB1 | XrivG2 | 0.009 |
| HS | XrivG2 | XrivY3 | 0.009 |
| HS | XrivB1 | XrivR2 | 0.009 |
| HS | XvGLA | XrivG2 | 0.012 |
| HS | XrivG1 | XrivR1 | 0.014 |
| HS | XvCHEL | XrivR2 | 0.014 |
| HS | XrivY3 | XrivR1 | 0.015 |
| HS | XrivB1 | XrivR1 | 0.016 |
| HS | XrivG2 | XrivR2 | 0.017 |
| HS | XrivG2 | XrivR1 | 0.031 |
| HS | XvGLA | XrivG1 | 0.048 |
| **LA** | **XrivR1** | **XrivR2** | **0.000** |
| LA | XrivG1 | XrivR2 | 0.021 |
| LA | XrivG1 | XvCHEL | 0.022 |
| SH | XvGLA | XvCHEL | 0.017 |
| SH | XvGLA | XrivY3 | 0.023 |
| SH | XrivG1 | XrivR2 | 0.037 |
| **ST** | **XrivR1** | **XrivR2** | **0.000** |
| ST | XvCHEL | XrivR1 | 0.035 |
| **TE** | **XvGLA** | **XvCHEL** | **0.000** |
| TE | XrivR1 | Xriv_2 | 0.002 |
| WI | XvCHEL | XrivR2 | 0.024 |
| WS | XrivG1 | XrivR2 | 0.030 |
