# Supplementary material for: Historical Environment Is Reflected in Modern Population Genetics and Biogeography of an Island Endemic Lizard (Xantusia riversiana reticulata)
Source: PLoS One. 2016 Nov 9;11(11):e0163738. doi: 10.1371/journal.pone.0163738 (PMC5102444; doi:10.1371/journal.pone.0163738)
Supplement: S5 Table — Allele counts and summary statistics for each locus (rows) by collection site (columns). (DOCX) [file pone.0163738.s007.docx]

S5 Table. Per locus and population allele counts. Allele counts and summary statistics for each locus (rows) by collection site (columns).

|  | **BO** | **EP** | **ES** | **HN** | **HS** | **LA** | **SC** | **SH** | **ST** | **TE** | **WI** | **WS** | **mean** | **s.d.** |
| --- | --- | --- | --- | --- | --- | --- | --- | --- | --- | --- | --- | --- | --- | --- |
| **XrivB1** | 1 | 1 | 2 | 2 | 2 | 1 | 1 | 1 | 1 | 1 | 1 | 1 | *1.25* | *0.43* |
| **XvGLA** | 14 | 16 | 15 | 18 | 22 | 16 | 8 | 16 | 21 | 23 | 17 | 11 | *16.42* | *4.15* |
| **XrivG2** | 1 | 1 | 2 | 2 | 4 | 1 | 1 | 1 | 1 | 1 | 2 | 1 | *1.50* | *0.87* |
| **XrivG1** | 7 | 12 | 8 | 10 | 10 | 7 | 3 | 6 | 9 | 8 | 9 | 7 | *8.00* | *2.20* |
| **XvCHEL** | 9 | 9 | 9 | 12 | 12 | 11 | 8 | 13 | 9 | 10 | 10 | 11 | *10.25* | *1.48* |
| **XrivY3** | 1 | 2 | 2 | 1 | 2 | 1 | 2 | 3 | 2 | 2 | 2 | 2 | *1.83* | *0.55* |
| **XrivR1** | 5 | 5 | 7 | 6 | 10 | 6 | 4 | 6 | 6 | 7 | 7 | 5 | *6.17* | *1.46* |
| **XrivR2** | 4 | 4 | 3 | 4 | 6 | 2 | 2 | 6 | 4 | 5 | 4 | 3 | *3.92* | *1.26* |
| **mean** | *5.25* | *6.25* | *6* | *6.875* | *8.5* | *5.625* | *3.625* | *6.5* | *6.625* | *7.125* | *6.5* | *5.125* | *6.17* | *1.16* |
| **s.d.** | *4.323* | *5.19* | *4.359* | *5.6* | *6.225* | *5.195* | *2.69* | *5.074* | *6.224* | *6.772* | *5.074* | *3.887* | *5.05* | *1.08* |
| **No. gene copies** | 64 | 72 | 62 | 126 | 248 | 76 | 22 | 72 | 74 | 70 | 72 | 74 | *86.00* | *53.41* |
| **No. total loci** | 8 | 8 | 8 | 8 | 8 | 8 | 8 | 8 | 8 | 8 | 8 | 8 | *8.00* | *0.00* |
| **No. usable loci** | 8 | 8 | 8 | 8 | 8 | 8 | 8 | 8 | 8 | 8 | 8 | 8 | *8.00* | *0.00* |
| **No. poly loci** | 5 | 6 | 8 | 7 | 8 | 5 | 6 | 6 | 6 | 6 | 7 | 6 | *6.33* | *0.94* |
