# Supplementary material for: Historical Environment Is Reflected in Modern Population Genetics and Biogeography of an Island Endemic Lizard (Xantusia riversiana reticulata)
Source: PLoS One. 2016 Nov 9;11(11):e0163738. doi: 10.1371/journal.pone.0163738 (PMC5102444; doi:10.1371/journal.pone.0163738)
Supplement: S6 Table — Sites in bold (10 of 12) are all within 5km of each other on the low-lying western side of the island characterized by high quality MSS habitat (see Fig 1b). (DOCX) [file pone.0163738.s008.docx]

S6 Table. Private alleles (*N*=12) by collection location. Sites in bold (10 of 12) are all within 5km of each other on the low-lying western side of the island characterized by high quality MSS habitat (see Fig 1b).

| **Site** | **Locus** | **Allele** | **Frequency** |
| --- | --- | --- | --- |
| **EP** | GLA | 350 | 0.014 |
| **ES** | XrivB1 | 132 | 0.032 |
| **HN** | XrivB1 | 125 | 0.048 |
| **HN** | XrivB1 | 114 | 0.008 |
| **HS** | GLA | 287 | 0.004 |
| **HS** | XrivG2 | 120 | 0.004 |
| **HS** | XrivG2 | 128 | 0.008 |
| **HS** | XrivR1 | 137 | 0.008 |
| **HS** | XrivR2 | 237 | 0.008 |
| SH | GLA | 291 | 0.014 |
| ST | GLA | 346 | 0.014 |
| **TE** | GLA | 344 | 0.014 |
