# Supplementary material for: Historical Environment Is Reflected in Modern Population Genetics and Biogeography of an Island Endemic Lizard (Xantusia riversiana reticulata)
Source: PLoS One. 2016 Nov 9;11(11):e0163738. doi: 10.1371/journal.pone.0163738 (PMC5102444; doi:10.1371/journal.pone.0163738)
Supplement: S7 Table — Number of migrants (Nm) per generation between pairs of collection sites. Migration rates higher than 5 are in bold. (DOCX) [file pone.0163738.s009.docx]

S7 Table. Pairwise numbers of migrants. Number of migrants (N_m_) per generation between pairs of collection sites. Migration rates higher than 5 are in bold.

|  | **BO** | **EP** | **ES** | **HN** | **HS** | **LA** | **SC** | **SH** | **ST** | **TE** | **WI** | **WS** |
| --- | --- | --- | --- | --- | --- | --- | --- | --- | --- | --- | --- | --- |
| **BO** | 0.000 |  |  |  |  |  |  |  |  |  |  |  |
| **EP** | 1.795 | 0.000 |  |  |  |  |  |  |  |  |  |  |
| **ES** | 2.042 | **52.829** | 0.000 |  |  |  |  |  |  |  |  |  |
| **HN** | 1.515 | 3.509 | 3.025 | 0.000 |  |  |  |  |  |  |  |  |
| **HS** | 1.337 | 2.273 | 2.044 | **23.835** | 0.000 |  |  |  |  |  |  |  |
| **LA** | 1.549 | 4.137 | 3.552 | **21.358** | **10.739** | 0.000 |  |  |  |  |  |  |
| **SC** | 1.355 | 4.869 | 4.143 | 1.773 | 1.271 | 2.124 | 0.000 |  |  |  |  |  |
| **SH** | 3.315 | 1.900 | 2.067 | 1.398 | 1.287 | 1.505 | 1.438 | 0.000 |  |  |  |  |
| **ST** | 2.337 | **7.127** | **7.957** | **5.814** | 4.049 | **8.695** | 4.595 | 2.113 | 0.000 |  |  |  |
| **TE** | 1.617 | **7.528** | **6.197** | **6.252** | 4.703 | **14.883** | 3.729 | 1.820 | **26.690** | 0.000 |  |  |
| **WI** | **8.001** | 2.311 | 2.345 | 2.352 | 2.258 | 2.467 | 1.761 | **8.650** | 3.432 | 2.591 | 0.000 |  |
| **WS** | 1.689 | 4.518 | 4.361 | 2.395 | 1.893 | 3.646 | **9.006** | 1.635 | **6.094** | **6.739** | 2.301 | 0.000 |
