# Supplementary material for: Historical Environment Is Reflected in Modern Population Genetics and Biogeography of an Island Endemic Lizard (Xantusia riversiana reticulata)
Source: PLoS One. 2016 Nov 9;11(11):e0163738. doi: 10.1371/journal.pone.0163738 (PMC5102444; doi:10.1371/journal.pone.0163738)
Supplement: S8 Table — (DOCX) [file pone.0163738.s010.docx]

S8 Table. Pairwise F_ST_ between all pairs of populations.

|  | **BO** | **EP** | **ES** | **HN** | **HS** | **LA** | **SC** | **SH** | **ST** | **TE** | **WI** | **WS** |
| --- | --- | --- | --- | --- | --- | --- | --- | --- | --- | --- | --- | --- |
| **BO** | 0 |  |  |  |  |  |  |  |  |  |  |  |
| **EP** | 0.139 | 0 |  |  |  |  |  |  |  |  |  |  |
| **ES** | 0.1223 | 0.0570 | 0 |  |  |  |  |  |  |  |  |  |
| **HN** | 0.1091 | 0.0658 | 0.0047 | 0 |  |  |  |  |  |  |  |  |
| **HS** | 0.1416 | 0.0116 | 0.0665 | 0.0763 | 0 |  |  |  |  |  |  |  |
| **LA** | 0.1575 | 0.0228 | 0.0991 | 0.1090 | 0.0104 | 0 |  |  |  |  |  |  |
| **SC** | 0.1558 | 0.1053 | 0.0488 | 0.0569 | 0.1236 | 0.1643 | 0 |  |  |  |  |  |
| **SH** | 0.0701 | 0.1424 | 0.1163 | 0.1079 | 0.1517 | 0.1627 | 0.1481 | 0 |  |  |  |  |
| **ST** | 0.0967 | 0.0280 | 0.0339 | 0.0305 | 0.0412 | 0.0582 | 0.0516 | 0.1058 | 0 |  |  |  |
| **TE** | 0.1339 | 0.0165 | 0.0321 | 0.0388 | 0.0385 | 0.0505 | 0.0628 | 0.1208 | 0.0093 | 0 |  |  |
| **WI** | 0.0303 | 0.0920 | 0.0976 | 0.0963 | 0.0961 | 0.0997 | 0.1243 | 0.0281 | 0.0679 | 0.0880 | 0 |  |
| **WS** | 0.1290 | 0.0641 | 0.0524 | 0.0542 | 0.0945 | 0.117 | 0.027 | 0.132 | 0.0394 | 0.0358 | 0.098 | 0 |
