# Supplementary material for: Historical Environment Is Reflected in Modern Population Genetics and Biogeography of an Island Endemic Lizard (Xantusia riversiana reticulata)
Source: PLoS One. 2016 Nov 9;11(11):e0163738. doi: 10.1371/journal.pone.0163738 (PMC5102444; doi:10.1371/journal.pone.0163738)
Supplement: S9 Table — (DOCX) [file pone.0163738.s011.docx]

S9 Table. Pairwise BayesAss measurements between all pairs of populations.

|  | **BO** | **EP** | **ES** | **HN** | **HS** | **LA** | **SC** | **SH** | | | **ST** | **TE** | **WI** | **WS** |
| --- | --- | --- | --- | --- | --- | --- | --- | --- | --- | --- | --- | --- | --- | --- |
| **BO** | 0.8305 | 0.0148 | 0.0071 | 0.0115 | 0.012 | 0.0082 | 0.0075 | | 0.0155 | 0.0382 | | 0.0094 | 0.013 | 0.0322 |
| **EP** | 0.0146 | 0.8404 | 0.0068 | 0.0176 | 0.0169 | 0.0073 | 0.0071 | | 0.0239 | 0.0247 | | 0.0119 | 0.0102 | 0.0186 |
| **ES** | 0.0159 | 0.1951 | 0.6747 | 0.0098 | 0.0136 | 0.0096 | 0.0098 | | 0.0123 | 0.0262 | | 0.0097 | 0.0092 | 0.014 |
| **HN** | 0.022 | 0.0365 | 0.0047 | 0.7141 | 0.1549 | 0.0056 | 0.0046 | | 0.0093 | 0.0246 | | 0.0061 | 0.0057 | 0.0119 |
| **HS** | 0.0036 | 0.0076 | 0.0035 | 0.0125 | 0.9094 | 0.0032 | 0.0033 | | 0.0125 | 0.0243 | | 0.0049 | 0.0053 | 0.0099 |
| **LA** | 0.0241 | 0.0128 | 0.0069 | 0.0149 | 0.1518 | 0.6735 | 0.0067 | | 0.0157 | 0.0191 | | 0.0075 | 0.0074 | 0.0598 |
| **SC** | 0.0192 | 0.0241 | 0.0146 | 0.0148 | 0.0188 | 0.0147 | 0.6813 | | 0.0207 | 0.1032 | | 0.0158 | 0.0225 | 0.0503 |
| **SH** | 0.0125 | 0.0746 | 0.0071 | 0.0221 | 0.0308 | 0.0079 | 0.0071 | | 0.7474 | 0.0285 | | 0.0108 | 0.0122 | 0.039 |
| **ST** | 0.0169 | 0.0198 | 0.0069 | 0.0292 | 0.064 | 0.0075 | 0.0064 | | 0.019 | 0.7831 | | 0.0102 | 0.0177 | 0.0194 |
| **TE** | 0.0087 | 0.0149 | 0.0074 | 0.0168 | 0.0663 | 0.0076 | 0.0068 | | 0.0136 | 0.0927 | | 0.6749 | 0.0093 | 0.081 |
| **WI** | 0.0173 | 0.0285 | 0.0068 | 0.013 | 0.0387 | 0.0073 | 0.0071 | | 0.0221 | 0.0426 | | 0.0083 | 0.6771 | 0.1313 |
| **WS** | 0.0627 | 0.0118 | 0.0068 | 0.009 | 0.013 | 0.0076 | 0.0068 | | 0.0325 | 0.0191 | | 0.0084 | 0.0141 | 0.8081 |
